# A clinical study to assess the effectiveness of a hyaluronic acid-based procedure for treatment of premature ejaculation

**DOI:** 10.1038/ijir.2013.13

**Published:** 2013-04-04

**Authors:** A Littara, B Palmieri, V Rottigni, T Iannitti

**Affiliations:** 1 Centro di Medicina Sessuale, Milan, Italy; 2 Istituto di Laser-Chirurgia Sessuale, Milan, Italy; 3 Department of General Surgery and Surgical Specialties, University of Modena and Reggio Emilia Medical school, Surgical Clinic, Modena, Italy; 4 Poliambulatorio del Secondo Parere, Modena, Italy; 5 Institute for Membrane and Systems Biology, University of Leeds, Leeds, UK

**Keywords:** augmentation, filler, glans, hyaluronic acid, penis

## Abstract

Premature ejaculation is a sexual debilitating condition affecting a large number of men worldwide and leading to important dysfunctions influencing the patients' affective and emotional life. Hyaluronic acid is a natural and safe compound that has been widely used not only in the aesthetic medicine clinic, but also for treatment of osteoarthritis. The present study shows the effectiveness of a hyaluronic acid-based procedure for treatment of premature ejaculation. A hundred and ten male patients were treated with hyaluronic acid injections in the deep dermis of their glans penis to increase the volume and the circumference of their penis to prevent male premature ejaculation and improve the patients' and their partners' sexual satisfaction. The intravaginal ejaculation latency time increased significantly from a baseline value of 88.34±3.14 s to 293.14±8.16 s after 6 months from the procedure. Maximal glandular circumference increased from 98.51±0.71 mm to 114.35±0.66 mm after 6 months from the procedure. At 6-month follow-up, patients' self-rated satisfaction was 5.3±0.07 (range: 4–6). At the follow-up, partners' self-rated satisfaction was 5.1±0.09 (range: 3–6). The present clinical study showed that hyaluronic acid injection is a promising treatment for premature ejaculation. The effect of the procedure in the long-term follow-up needs to be clarified.

## Introduction

Premature ejaculation (PE) is a common sexual debilitating dysfunction affecting 20–30% of all men worldwide.^1, 2^ A definition of PE has not been standardized yet^3^ because of the large and controversial debate surrounding this term. However, in medical practice, this term is commonly defined as ‘the ejaculation that regularly occurs at or around initial vaginal penetration',^4^ and it is not the result of a prolonged absence from sexual activity.^5^ To achieve a precise diagnosis, the specialist often relies only on the patient's history without further laboratory or physiological tests.^1^ PE pharmacological treatment includes the use of antidepressants, local anesthetic agents and phosphodiesterase type 5 inhibitors; currently, dapoxetine represents the only short-acting selective serotonin reuptake inhibitor licensed for PE treatment.^1^ Filler materials have been extensively used for soft tissue augmentation in aesthetic surgery, but it is only recently that they have found an important application in the field of glans penis augmentation allowing to achieve a volume sufficient to prevent PE.^2, 6^ Abdallah *et al.*^2^ evaluated the effect of hyaluronic acid in 60 men affected by PE, demonstrating that intravaginal ejaculation latency time (IELT) increased 1 month after the injection of the compound in their penis. Furthermore, in 2008, another study^6^ reported positive results in a 5-year long-term study in which hyaluronic acid gel was injected in 38 men. They demonstrated that IELT decreased if compared to 6-month follow-up, but it was still higher if compared with the pretreatment period. The patients and their partners reported high satisfaction for the procedure consisting in 76% and 63%, respectively.

### Aim

The aim of this study was to investigate the effectiveness of hyaluronic acid injection in the deep dermis of the glans penis to increase the volume and the circumference of the penis to prevent male PE and improve the patients' and their partners' sexual satisfaction. The rationale for this procedure is to increase the dermis thickness of the glans embedding the nervous fibres in highly cross-linked hyaluronic acid atmosphere to reduce sensitivity of penile reflexes.

## Materials and methods

### Patients

A total of 171 male patients were screened between June 2009 and March 2011. A hundred and ten male patients, aged between 25 and 42 years (32.78±0.33; mean±standard error of the mean (s.e.m.)), affected by PE, were included in this study. For the purposes of the present investigation, PE was defined as involuntary ejaculation during foreplay or within 1 min of penetration on at least 50% of occasions when attempting intercourse.^7^ Patients were selected at our clinic where the study was performed. Inclusion criteria were a stable, monogamous and heterosexual relationship for at least 12 months. Exclusion criteria were a history of medication that can affect ejaculation 6 months before the beginning of the study, a history of drug abuse within 2 years before enrollment for the procedure, a history of or current major psychiatric disorder (psychiatric consultation was conducted at the time of enrollment), such as mood and anxiety disorders, schizophrenia, other psychotic disorders, alcoholism, erectile dysfunction and patients' or partners' decreased interest in sexual intercourse or other forms of sexual dysfunction. No other medication of psychotherapy was allowed during the study period.

### Surgical procedure

**Figure 1:**
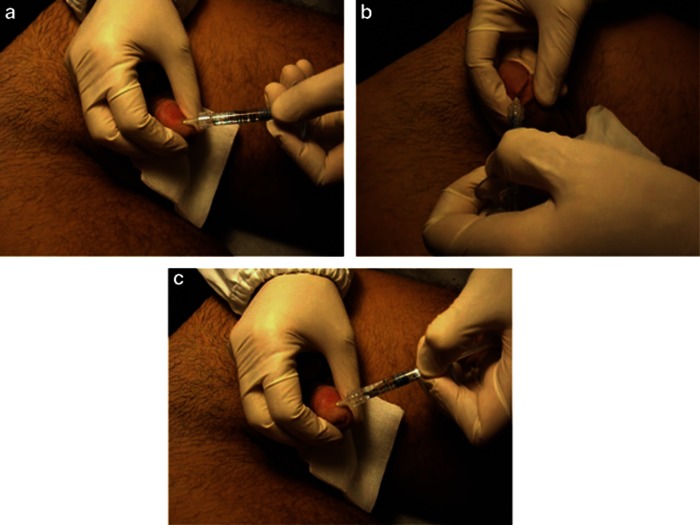
(**a**–**c**) The circumference of the glans penis is virtually subdivided into three circles (starting from the base of the glans penis at a distance of 1 cm from each other). Then, each circumference is further subdivided into quarter circles where 1 ml of hyaluronic acid is injected.

The present study was performed according to the Helsinki declaration and local internal review board approval was obtained (it is available for viewing upon request from the Editor-in-Chief). All patients signed the informed consent. Each patient, comfortably sitting, was injected with a 28-G needle containing 1 ml of 1% lidocaine and prilocaine (Astra Pharmaceuticals, Milan, Italy) to induce local anesthesia. The circumference of the glans penis (deep connective tissue of the corpus spongiosum) was divided into three circles (from the base of the glans at a 1-cm distance from each other). The circles were then divided into quarter circles. An injection, containing 1 ml hyaluronic acid (Variofill; Adoderm GmbH, Langenfeld, Germany) was performed in the deep dermis into every quarter circle with a 27-G needle for a total of 12 injections performed in a single session (Figure 1). The patients' and partners' satisfaction was rated on the basis of a scale 1–10 (1=dissatisfied; 10=satisfied). IELT, a subjective measure defined as the time between the start of vaginal intromission and the start of intravaginal ejaculation, was evaluated for each couple. Evaluations about the increase of glans circumference and patients' and partners' satisfaction were also performed. Following the procedure, the patients did not receive any further aesthetic treatment.

Patients and partners were asked to self-rate their satisfaction with sexual intercourse before and after the procedure, using a scale from 1 to 6 (1=not satisfied; 6=very satisfied). This information was collected by an allied health-care professional and kept in our clinic database.

### Statistical analysis

All data are represented as the means±s.e.m., and were first checked for normality using the Anderson–Darling test. All statistical analysis was conducted using Minitab, v15, Leeds, UK. A paired *t*-test was used to compare IELT and maximal glandular circumference before and after treatment. A value of *P*<0.05 was considered significant.

## Results

**Figure 2:**
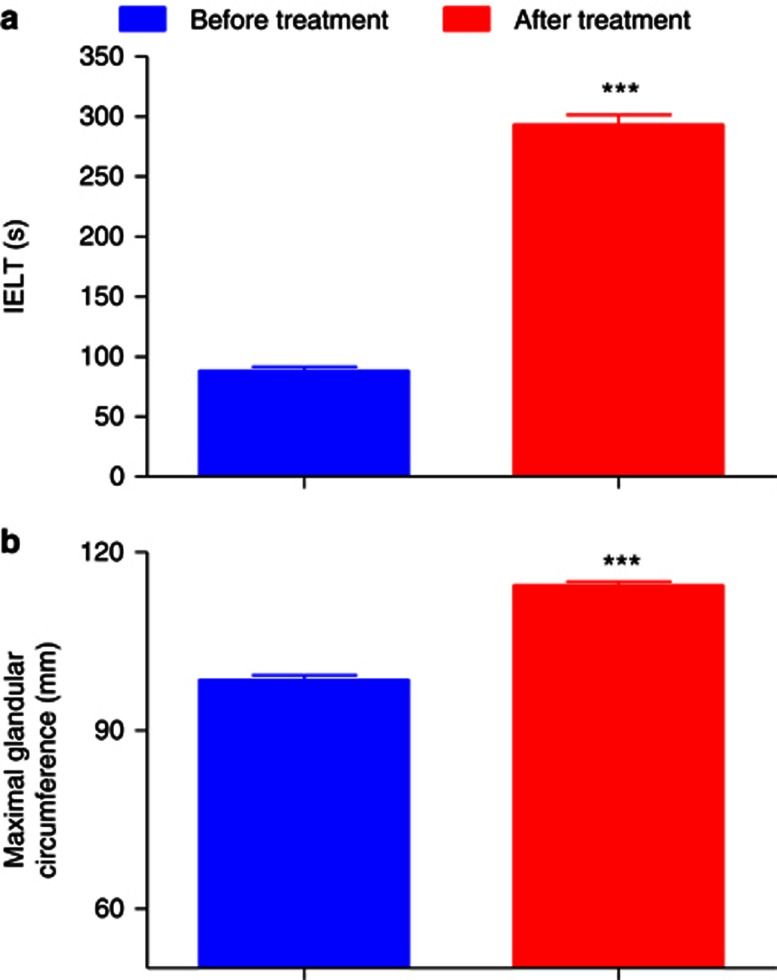
(**a**). IELT (s) and (**b**) maximal glandular circumference (mm) before and after surgical procedure. Data are presented as the group mean±s.e.m. ^***^P<0.001.

**Figure 3:**
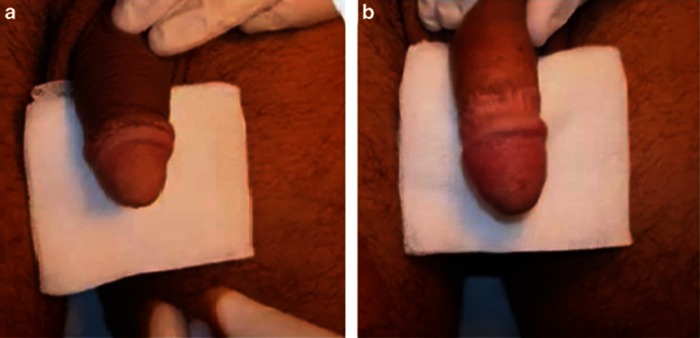
Clinical situation (**a**) before and (**b**) after hyaluronic acid injection in the glans penis.

**Figure 4:**
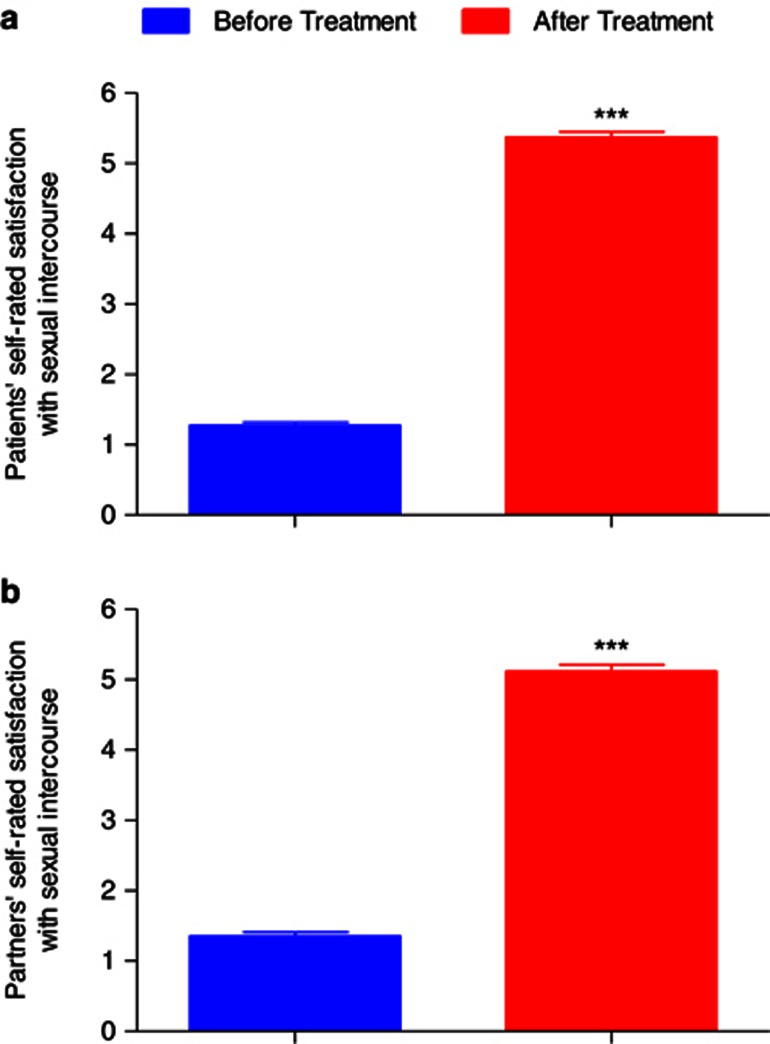
Patients' (**a**) and partners' (**a**) self-rated satisfaction with sexual intercourse before and after surgical procedure. Data are presented as the group mean±s.e.m. ***P<0.001.

At baseline, patients' self-rated satisfaction with sexual intercourse was 1.2±0.04 (Figure 4a). Partners' self-rated satisfaction with sexual intercourse was 1.3±0.05 (Figure 4b). Treatment was well tolerated. No dropout or uncompleted procedure was reported. No pain was observed when performing local anesthesia. No inflammatory signs or other adverse reactions were observed in all cases. The IELT increased significantly from 88.34±3.14 s to 293.14±8.16 s after 6 months from the procedure (*P*<0.001; Figure 2a). Maximal glandular circumference, measured by tapeline, increased from 98.51±0.71 mm as measured before treatment, to 114.35±0.66 mm at 6 months (*P*<0.001; Figure 2b; Figure 3). At 6-month follow-up, patients' self-rated satisfaction with sexual intercourse was 5.3±0.07 (*P*<0.001; Figure 4a). At the follow-up, partners' self-rated satisfaction with sexual intercourse was 5.1±0.09 (*P*<0.001; Figure 4b).

## Discussion and Conclusions

The present study shows that hyaluronic acid injection can be effectively used for treatment of PE, allowing to achieve a significant increase in IELT. At 6-month follow-up, IELT was still significantly higher, if compared with baseline values. The maximal glandular circumference was significantly increased at 6-month follow-up. Self-rated patients' and partners' sexual satisfaction was rated as 5 or 6 by 90 and 74 subjects, respectively, at 6-month follow-up. According to the present study, the procedure is well tolerated without adverse reactions. This article describes an original hyaluronic acid-based approach to treat PE and well confirms the favourable outcome previously reported in an experimental study in rabbits and dogs^8^ where hyaluronic acid was injected into the glans penis proving its potential for glandular augmentation. In fact hystological analysis showed that hyaluronic acid can still be found in the lamina propria of the glans penis after 6 months.^8^ Another two studies performed in men by the same research group,^9, 10^ also support the use of hyaluronic acid as a safe and effective approach for glans penis augmentation and treatment of PE. Findings from our study also support the use of hyaluronic acid for treatment of PE. Further studies, with a follow-up extending beyond 6 months, are necessary to determine with precision the long-term therapeutic capacity of this treatment. Hyaluronic acid has been widely used in aesthetic surgery, and complications are very rare and promptly manageable by expert surgeons. Therefore, it is possible that such a procedure, based on the protocol we are proposing, may be integrated in the aesthetic clinic and performed on a routine basis.
